# Automatic versus manual tuning of robot-assisted gait training in people with neurological disorders

**DOI:** 10.1186/s12984-019-0630-9

**Published:** 2020-01-28

**Authors:** Simone S. Fricke, Cristina Bayón, Herman van der Kooij, Edwin H. F. van Asseldonk

**Affiliations:** 1 Department of Biomechanical Engineering, University of Twente, Enschede, The Netherlands; 2 Department of BioMechanical Engineering, Delft University of Technology, Delft, The Netherlands

**Keywords:** Robotic gait training, Rehabilitation, Gait, Stroke, Spinal cord injury, Assist-as-needed

## Abstract

**Background:**

In clinical practice, therapists choose the amount of assistance for robot-assisted training. This can result in outcomes that are influenced by subjective decisions and tuning of training parameters can be time-consuming. Therefore, various algorithms to automatically tune the assistance have been developed. However, the assistance applied by these algorithms has not been directly compared to manually-tuned assistance yet. In this study, we focused on subtask-based assistance and compared automatically-tuned (AT) robotic assistance with manually-tuned (MT) robotic assistance.

**Methods:**

Ten people with neurological disorders (six stroke, four spinal cord injury) walked in the LOPES II gait trainer with AT and MT assistance. In both cases, assistance was adjusted separately for various subtasks of walking (in this study defined as control of: weight shift, lateral foot placement, trailing and leading limb angle, prepositioning, stability during stance, foot clearance). For the MT approach, robotic assistance was tuned by an experienced therapist and for the AT approach an algorithm that adjusted the assistance based on performances for the different subtasks was used. Time needed to tune the assistance, assistance levels and deviations from reference trajectories were compared between both approaches. In addition, participants evaluated safety, comfort, effect and amount of assistance for the AT and MT approach.

**Results:**

For the AT algorithm, stable assistance levels were reached quicker than for the MT approach. Considerable differences in the assistance per subtask provided by the two approaches were found. The amount of assistance was more often higher for the MT approach than for the AT approach. Despite this, the largest deviations from the reference trajectories were found for the MT algorithm. Participants did not clearly prefer one approach over the other regarding safety, comfort, effect and amount of assistance.

**Conclusion:**

Automatic tuning had the following advantages compared to manual tuning: quicker tuning of the assistance, lower assistance levels, separate tuning of each subtask and good performance for all subtasks. Future clinical trials need to show whether these apparent advantages result in better clinical outcomes.

## Background

Robot-assisted gait training (RAGT) is a promising technique for rehabilitation after neurological disorders such as stroke or spinal cord injury (SCI). RAGT can be used to provide intensive, repetitive and task-specific training, while it also contributes to reduce physical load for therapists [1]. Reviews of previous studies have shown that RAGT can increase the likelihood that people walk independently after stroke, and that it is most effective in the acute phase after stroke/SCI and in the most impaired patients [2, 3]. However, those results should be handled with some care as differences in patient groups, robotic gait trainers, protocol guidelines and control algorithms can largely affect the outcomes [2, 4].

Regarding protocol guidelines and control algorithms, it has to be considered that the amount of assistance that the robotic gait trainers provide to the users is often manually tuned by therapists or cannot be changed [5–7]. Therapists mainly base their decisions on visual assessments of the patient, which means that training outcomes can be influenced by subjective decisions. Some studies address this issue by defining guidelines on how to set the assistance [6–9]. However, these guidelines are often not really specific and require experienced therapists to adjust training parameters.

Therapists might have difficulties while tuning the assistance for RAGT compared to manually assisted gait training (where therapists use their hands to move patient’s legs) due to two main reasons. First, in RAGT, therapists cannot directly feel the assistance that is being applied, and have to rely on other feedback (e.g. visual assessment of the patient) to choose the best assistance for the patient’s needs. Second, the large number of parameters to tune the provided amount/timing of assistance [10], makes it difficult and time-consuming to manually change them while observing the patient [4]. Therefore, manually-tuned controllers that are currently used for therapy have their limitations in tailoring therapy to the patients’ needs.

To objectively and quickly tune the robotic assistance and to promote active participation of the patient, various algorithms that automatically adjust the amount of robotic assistance for lower limbs [11–21] or upper limbs [22–26] have been developed. Some of these algorithms gradually adapt the assistance based on an error compared to a reference trajectory and a forgetting factor [13, 14, 16, 21]. Others use reference trajectories (e.g. for the hip and knee angle during walking) with an (adaptive) virtual tunnel around these trajectories [11, 12, 25]. Forces are applied by the device to prevent that the user moves out of the tunnel (i.e. too large deviations of joint angles compared to the reference trajectories). Most of these algorithms can tune the robotic assistance automatically and quickly at a joint level for each percentage of the gait cycle. However, they do not explicitly consider the different subtasks of walking (in this study defined as control of: weight shift, lateral foot placement, trailing and leading limb angle, prepositioning, stability during stance, foot clearance) [10, 27–30].

We previously developed an algorithm that is focused on these functional subtasks of gait and automatically tunes the amount of robotic assistance for each subtask based on the user’s performance during walking [15, 31]. This algorithm is designed to tune the assistance in a similar way as therapists would like to tune robotic assistance: judging which subtasks of gait are affected and applying assistance for these subtasks [32].

So far, automatically-tuned (AT) algorithms have mainly been evaluated in single sessions (e.g. effect on kinematics or EMG) [12, 16] or studies with a low number of participants [11, 33] while various larger clinical studies compared manually-tuned (MT) RAGT to conventional physical therapy [2]. As far as we know, the amount of robotic assistance applied by an AT algorithm has not been compared yet to the settings that a therapist would use and it is unknown how these two approaches affect rehabilitation in people with neurological disorders.

In the present, exploratory study, as a first step in getting more insight into the effect of MT and AT robotic assistance, we compare two different approaches for tuning robotic assistance by using the LOPES II gait trainer [10]: (1) subtask-based assistance set by an experienced therapist (manually-tuned, MT); and (2) subtask-based assistance set by our above-mentioned algorithm (automatically-tuned, AT) [15, 31]. By performing this comparison, we expect to answer the following questions: (1) How is the assistance tuned by the MT and AT approaches? (e.g. how long does it take to tune the assistance?); (2) Which final assistance levels are chosen for the MT and AT approach?; (3) How do these assistance levels affect deviations from the reference trajectories at specific evaluation points for each subtask (e.g. maximal hip and knee flexion)?; (4) Do the participants prefer one of the approaches over the other one regarding safety, comfort, effect and amount assistance?

The results from this study give more insight into how the two approaches, AT and MT assistance, affect RAGT and may be used to further optimize robot-based rehabilitation of patients with neurological disorders.

## Methods

### Participants

Six stroke survivors and four people with incomplete SCI, all in the chronic phase (>6 months after injury), participated in this study (7 male, age 53 ±17 years, weight 78 ±12 kg, height 1.76 ±0.12 m). An overview of the participants’ characteristics can be found in Table 1. Inclusion criteria used in this study were (1) age >18 years, (2) a stable medical condition, (3) a physical condition which allowed for 3 min of supported walking, (4) sufficient cognitive abilities to follow the instructions and report any discomfort, (5) time since stroke/SCI >6 months. People with other orthopedic or neurological disorders or cardiac conditions that could be affected by physical load were excluded.

**Table 1:** Overview of participant characteristics, clinical scores and settings for LOPES II

ID	Gender	Age	Time post stroke/SCI (years)	More affected leg	Level SCI	10MWT (km/h)	Assistive device(s) used during 10MWT	FAC	FMA	MI	Walking speed in LOPES II (km/h)	Toe-lifter in LOPES II
Str1	m	53	1	r	n.a.	1.9	cane, AFO	4	13	28	1.5	Yes
Str2	f	63	5	l	n.a.	2.1	cane	5	33	83	2.0	No
Str3	m	60	2	l	n.a.	2.4	cane, AFO	5	19	42	1.5	Yes
Str4	m	33	10	r	n.a.	3.1	none	5	25	67	1.4	No
Str5	m	74	4	r	n.a.	4.1	none	5	31	91	1.7	No
Str6	f	74	6	r	n.a.	3.0	none	5	24	58	1.5	No
SCI1	f	45	8	l	T9	2.8	walker	5	n.a.	n.a.	1.3	No
SCI2	m	25	3	l	L3	n.a.	n.a.	0	n.a.	n.a.	1.5	Yes
SCI3	m	63	6	r	T12	0.8	walker, AFO	3	n.a.	n.a.	1.0	Yes
SCI4	m	41	1	r	L3	2.9	none	5	n.a.	n.a.	2.0	No

ID: identification code used for each specific participant, 10MWT: 10 meter walking test, FAC: functional ambulation scale, FMA: Fugl-Meyer assessment (lower extremity), MI: Motricity index (lower extremity), AFO: ankle foot orthosis, Str..: participants with stroke, SCI..: participants with SCI

The experiments were approved by the local medical ethical committee (METC Twente) in accordance with the guidelines of the Declaration of Helsinki. All participants received oral and written information about the experiments and gave written informed consent prior to the start of the experiments.

### Robotic gait trainer

LOPES II (LOwer extremity Powered ExoSkeleton II) was used to evaluate the AT and MT approach in this study. LOPES II is a gait trainer consisting of push-pull rods which are attached to the pelvis and lower limbs of the user [10]. LOPES II can provide assistance for eight degrees of freedom (DOFs) (pelvis front/back, pelvis left/right, hip flexion/extension, hip abduction/adduction and knee flexion/extension) while the user is walking on an instrumented treadmill. LOPES II is an admittance-controlled device and the amount of robotic assistance can be set from minimal impedance (transparent mode, minimizing interaction forces between the device and human) to full assistance (mimicking position control). When applying assistance, LOPES II can move the user along different reference trajectories. The reference trajectories are defined for each DOF and are based on a data set from healthy elderly subjects [34]. The exact amount of force/torque that is applied to move the user along the reference trajectories depends on: (1) deviations from the reference trajectories and (2) stiffness *K* of virtual springs with equilibrium positions on the reference trajectories. This virtual spring stiffness *K* can be calculated with the following equation for each DOF (*j*) and each instant (*i* in %) of the gait cycle: $K_{j,i}=K_{max,j} \left (\frac {G_{j,i}}{100}\right)^{2}$. *K*_*max*,*j*_ is a maximal stiffness that is defined for each DOF of LOPES II (see [10]) and *G*_*j*,*i*_ is the desired assistance that is either MT or AT in this study. More details about the design and control of LOPES II can be found in [10].

#### Subtask-based assistance

The gait cycle was divided into various subtasks that are relevant for walking [10] (see Table 2 for an overview of the subtasks). Specific assistance profiles were used to assist when needed only at the portion of the gait cycle corresponding to each specific subtask (see Table 2). The subtask-based assistance could be adjusted individually, and separately for each leg. For example, left hip flexion could be assisted during swing to improve the leading limb angle on that side, while all other subtasks were in minimal impedance mode. As previously indicated, the assistance for each subtask was either chosen by a therapist (MT) or automatically calculated by the algorithm described below.

**Table 2:** Overview of subtasks

Subtask (affected DOF: evaluation point(s))	Reference and measured trajectories, evaluation points	Stiffness profile, reference trajectory	Calculation of error	Lower and upper bound
**Weight shift** (lateral pelvis position: minimal and maximal position)			*P*_*err*_=*max*(|*P*_*ref*,*e*1_−*P*_*meas*,*e*1_|, |*P*_*ref*,*e*2_−*P*_*meas*,*e*2_|)	2.50cm 4.17cm
**Lateral foot placement** (hip abduction angle: angle at 100% of gait cycle)			*θ*_*err*_=∣*θ*_*ref*,*e*1_−*θ*_*meas*,*e*1_∣	1.15deg. 1.91deg.
**Leading limb angle** (hip flexion angle: maximal angle)			*θ*_*err*_=*θ*_*ref*,*e*1_−*θ*_*meas*,*e*1_	2.15deg. 3.58 deg.
**Trailing limb angle** (hip flexion angle: minimal angle)			*θ*_*err*_=*θ*_*meas*,*e*1_−*θ*_*ref*,*e*1_	1.75deg. 2.92deg.
**Prepositioning** (knee flexion angle: angle at 100% of gait cycle)			*θ*_*err*_=*θ*_*meas*,*e*1_−*θ*_*ref*,*e*1_	4.29deg. 7.16deg.
**Stability during stance** (knee flexion angle: maximal angle between 10 and 40% of gait cycle)			*θ*_*err*_=∣*θ*_*ref*,*e*1_−*θ*_*meas*,*e*1_∣	4.30deg. 7.16deg.
**Foot clearance** (knee flexion angle: maximal angle)			*θ*_*err*_=*θ*_*ref*,*e*1_−*θ*_*meas*,*e*1_	4.52deg. 7.54deg.

**Fig. 1:**
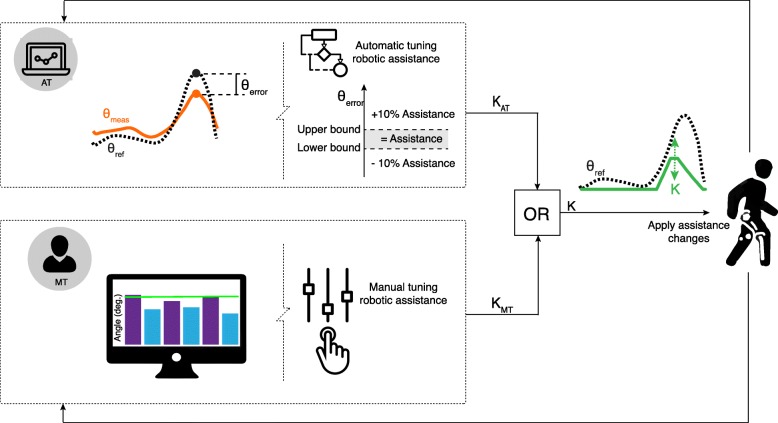
Overview of assistance tuning. The assistance was either AT based on the error between reference and measured trajectories or MT by a therapist. In this figure only an example for the foot clearance subtask is shown, however, the algorithm was applied to all subtasks shown in Table 2 simultaneously. For the AT algorithm, based on the error, every three steps, the assistance was either increased (if error >upper bound, see Table 2), decreased (if error <lower bound) or kept constant (other cases) by scaling the amplitude of the assistance profile (K) shown on the right. For the MT approach, the therapist could change the assistance (amplitude of the assistance profile K on the right) for each subtask by using graphical sliders. Feedback for the therapist was also shown to assist the therapist in tuning the assistance. As shown in this figure, the therapist got feedback about the maximal knee angle for the foot clearance subtask. The purple bars represented the maximal knee flexion angles for the previous three steps of the less impaired leg, while the blue bars represented the maximum knee flexion angles for the more impaired leg. The green line indicated the maximal knee flexion angle for the reference trajectory

Reference (black dotted lines) and measured (orange lines) positions and joint angles (P_ref_, P_ref_, *θ*_ref_, *θ*_ref_), assistance profiles (K) and evaluation points (e.g. P_ref,e1_) that were used to calculate the error are shown. Each figure shows one gait cycle starting with left heel strike at 0%. Weight shift to the right side, abduction and flexion angles are defined positive. The lower and upper bound are thresholds for adjusting the assistance based on the calculated error with the AT algorithm. If the error was lower than the lower bound, assistance was decreased. An error larger than the upper bound led to an increase in assistance and in other cases the assistance remained constant (see also Fig. 1)

#### Manually-tuned (MT) assistance

A graphical user interface (GUI) was used by an experienced physical therapist to set the amount of robotic assistance [10] (see Fig. 1). The GUI consisted of one main tab and one tab for each subtask. The subtask-based assistance could be adjusted individually with a minimal change of 10% by using a slider in the respective tab of the GUI. In addition, assistance levels for (all) subtasks could be coupled and the assistance levels for all coupled subtasks could be changed simultaneously by using a slider in the main tab of the GUI. To assist in tuning and show the immediate effects of changing assistance levels, visual feedback about the performance was provided for each subtask in the respective tab of the GUI (e.g. maximal knee flexion was shown for the foot clearance subtask, see Fig. 1). In this study, the same therapist, who was experienced in using LOPES II, tuned the amount of assistance for all experiments. The therapist got the instruction to set the assistance to a level that he would have used to train the patient. We decided not to give him more specific instructions as we were interested in which levels a therapist would choose without receiving any additional instructions.

#### Automatically-tuned (AT) assistance

The AT algorithm adjusted the amount of assistance based on the user’s performance [15, 31] (see Fig. 1). Specific evaluation points were defined for each subtask of walking (see Table 2). The reference and measured joint angles were determined for each evaluation point and the error was calculated as defined in Table 2. For some subtasks (foot clearance, trailing and leading limb angle, prepositioning), we assumed that exceeding the reference trajectory would not be detrimental. For example, we allowed maximum knee flexion larger than the reference gait pattern for the foot clearance subtask as too much knee flexion during swing is not typically found in people with stroke or SCI. In addition, the reference trajectories that were used in LOPES II are based on average trajectories of healthy individuals and might not exactly fit the needs of the user (with stroke or SCI). Allowing more knee flexion during swing (more foot clearance) than the reference pattern is safer as the feet will less likely hit the ground prematurely in the swing phase. For the same subtask, a knee flexion smaller than the reference pattern was penalized. For other subtasks (weight shift, stability during stance, lateral foot placement), we calculated the absolute error since an error in both directions might have negative consequences in people with neurological disorders. For example, during stance phase (subtask: stability during stance), both, knee hyperextension or too much knee flexion, can be found in people with neurological disorders [35].

Lower and upper bounds were defined for the subtask-based assistance based on the variability in the evaluation points in healthy participants walking in LOPES II in minimal impedance mode (see Table 2) [15]. After three steps, the average error per subtask and side was calculated to adjust the amount of robotic assistance for each subtask and side separately. The subtask-based assistance was increased by 10% if the average error was larger than the upper bound (see Fig. 1), as the user needed more assistance to stay closer to the reference trajectory. If the average error was lower than the lower bound, the amount of assistance was decreased by 10% to prevent that the user only relied on the assistance and to promote active participation. If the error was in between the lower and upper bound, the robotic assistance was kept constant.

### Experimental procedures

Each participant took part in two sessions (familiarization and experimental session) on two different days. The familiarization session was used to gather information about the participants (e.g. clinical scores) and practice walking in LOPES II. The experiments to compare AT and MT assistance were performed in the experimental session.

In the familiarization session, clinical tests (10 meter walking test (10MWT), Functional Ambulation Category (FAC), Fugl-Meyer assessment (FMA), Motricity index (MI)) were administered by a therapist. After this, participants’ upper and lower leg lengths and pelvis width was measured and adjusted in the software and hardware settings of LOPES II. Participants were strapped into LOPES II and toe-lifters were attached if participants dragged their toes along the ground during the swing phase. Participants with stroke, if needed, only used a toe-lifter on the more impaired side while participants with SCI used toe-lifters for both feet. Walking speed and, if needed, partial body weight support (PBWS) was set to a comfortable value based on the feedback from the participant and therapist (see Table 1). To get used to walking in LOPES II, participants walked at least two times, for three minutes in the device in this familiarization session. The first time, the assistance was set manually while the second time the AT algorithm was used to allow the user to experience both approaches. Participants were allowed to use the handrails of LOPES II during both sessions.

In the experimental session, the same settings (walking speed, PBWS, toe-lifters) as in the familiarization session were used to assess the AT and MT approach. Each participant performed four trials: MT_var_, MT_const_, AT_var_ and AT_const_ (var: variable assistance during the trial, const: constant assistance, as described below and in Table 3). Half of the participants started with MT assistance (MT_var_, MT_const_) and the other half started with AT assistance (AT_var_, AT_const_). Between the different trials, participants could take breaks. If needed, a break could be taken during MT_var_. If AT_const_ or MT_const_ was getting too exhausting for the participants, they could stop after less than three minutes. For both approaches, participants with a FAC score larger than 3, started at 30% of robotic assistance (following our clinical partner’s advise), all other participants started at 100% assistance for all subtasks.

**Table 3:** Overview of the trials of the experimental session

Trial name	Duration	Assistance
MT_var_	As much as therapist needed	Manually tuning assistance (therapist), variable (var) assistance during the trial
MT_const_	3 min	Constant (const) at level that therapist chose
AT_var_	3 min	Automatically tuning assistance, variable (var) assistance during the trial
AT_const_	3 min	Constant (const) at level of last 15 steps of AT_var_

Each participant took part in all trials. Half of the participants started with the MT trials, while the other half started with the AT trials

In MT_var_, the therapist set the amount of assistance using the GUI. While tuning the assistance, the therapist was able to visually assess the gait pattern and to get verbal feedback from the participant by talking to him/her. The therapist also received visual feedback about the performance for each subtask in the GUI. The therapist could take as much time as needed to set the robotic assistance to a final level that he/she would use for a training session with the specific participant. Subsequently, in MT_const_, the assistance was kept constant at the final assistance levels that the physical therapist had chosen in MT_var_. Participants walked for three minutes with these settings.

In AT_var_, participants walked for three minutes with the adaptive AT algorithm, which automatically adjusted the amount of robotic assistance based on users’ performance as explained in the previous section. After three minutes, LOPES II was stopped. Subsequently, in AT_const_, participants walked for three minutes while keeping the subtask-based assistance constant at the average assistance levels calculated with the last 15 steps of AT_var_ (rounded to the nearest tens).

### Outcome measures

To analyze differences between the AT and MT approach, we focused on different aspects that are described in this section: assistance tuning, final amount of assistance, errors at final amount of assistance, PBWS and questionnaires that were filled in by the participants and the therapist.

#### Assistance tuning

The time at which a stable assistance level was reached, was determined for each participant and each subtask for AT_var_ and MT_var_. The AT algorithm might change the assistance by 10% every three steps, never reaching a completely stable level. Therefore, it was defined that a stable level was reached when no changes larger than 10%, compared to the final assistance level of the trial, occurred. A two-sided Wilcoxon signed rank test was used to evaluate differences in the time that was needed to tune the assistance. A p-value lower than 0.05 was considered significant.

#### Final assistance levels

The applied robotic assistance was compared between AT_const_ and MT_const_ for each participant and each subtask of walking.

#### Errors for final assistance levels

The average error (difference between reference and measured trajectory) for AT_const_ and MT_const_ was calculated for each participant and subtask. In the results section we focus on the errors above the upper bounds (negative effects on participant’s gait), which are defined in Table 2.

#### Partial body weight support

Participants were allowed to use the handrails during walking and might have varied the amount of force applied to the handrails to support their own weight. To make sure that there were no large differences in the amount of PBWS between the MT and AT trials, the average PBWS was calculated by using the vertical forces measured with the force sensors under the walking surface of the treadmill.

#### Questionnaires

##### Participants’ preferences:

Participants filled out a self-administered paper-based questionnaire about the trials with MT and AT assistance. The questionnaire contained the following four questions that were evaluated, for each approach, on a scale from 1 to 5 (1 being very unsatisfied and 5 being very satisfied):

*How satisfied are you with...*

1. *...the safety experienced in the robot (do you feel safe)?*
2. *...the comfort during walking in the robot (assistance or resistance)?*
3. *...the effect of assistance on walking in the robot?*
4. *...the amount of assistance given by the robot?*

Average scores and standard deviations were calculated for each question that participants filled in.

##### Therapist:

To get more insight into how the therapist was choosing the assistance provided by LOPES II, the therapist filled in a short questionnaire with the following two questions:

1. *Which settings did you adjust and why?*
2. *Are you satisfied with the result? Why (not)? (For example, were there things that you could not change in the way you wanted?)*

In this paper, only the most common answers are reported and we do not focus on specific answers that were only given for a small number of participants.

## Results

All participants were able to perform the protocol and walk with the AT and MT algorithm. However, for SCI2, AT_const_ was stopped after two minutes (instead of three minutes) as the participant was getting too exhausted.

### Assistance tuning

On average, a stable assistance level for MT_var_ (difference to final level <10% for all subtasks) was reached after 279 ±120 sec. For AT_var_, a stable level was reached more quickly (after 110 ±54 sec.). The Wilcoxon signed rank test indicated that this difference between the MT and AT approach was significant (Z =-3.60, *p* =0.006).

**Fig. 2:**
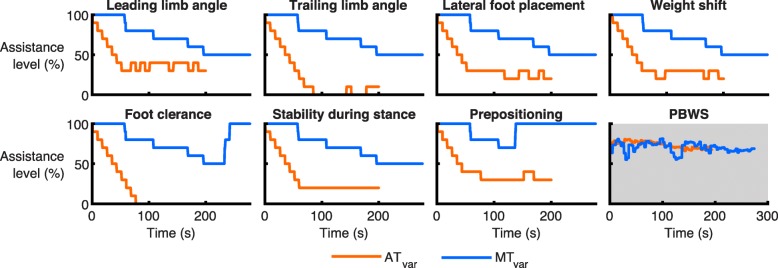
Assistance levels while tuning the assistance in SCI3. Assistance levels for all subtasks of the more impaired leg and weight shift are shown for AT_var_ and MT_var_ of participant SCI3. The subfigure with grey background shows the measured PBWS (provided by LOPES II and use of the handrails by the participant)

For the AT approach, in the beginning of the trial, the assistance for each subtask was changed every three steps until it approached its final stable level (changes of maximal 10%). In contrast to this, the therapist (MT approach) often focused on decreasing the assistance for all subtasks simultaneously (i.e. coupling all subtasks in the GUI) and then increasing the assistance for (one to four) specific subtasks. As an example, Fig. 2 shows these differences in tuning the assistance for the hip and knee flexion of one participant (SCI3).

### Final assistance levels

**Fig. 3:**
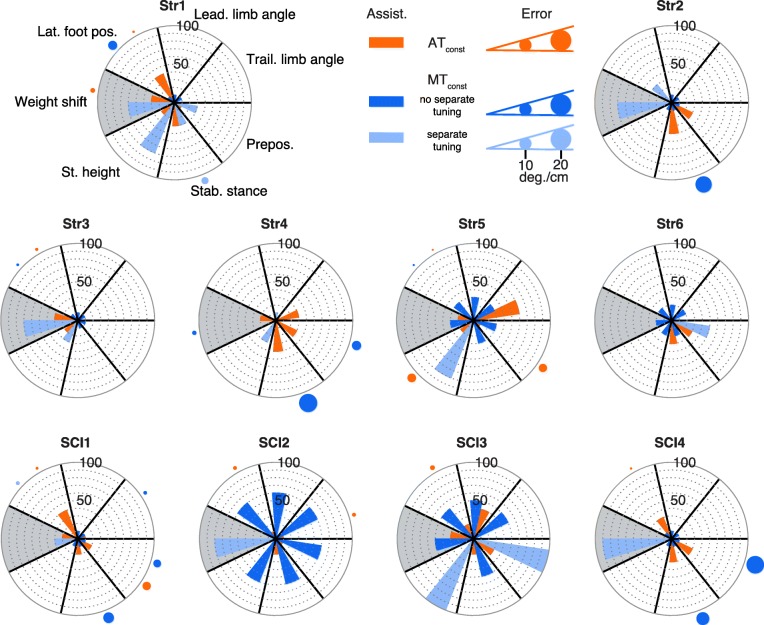
Assistance and errors for AT_const_ and MT_const_ for the more impaired leg (white background) and weight shift (grey background). Each polar plot shows the results for one participant. The distribution of the subtasks is the same for all polar plots (see Str1). The results for MT_const_ are split up into subtasks that were separately tuned by the therapist in a specific participant (light blue) and subtasks that were not separately tuned (dark blue). The bars represent the amount of assistance that the participants received for each specific subtask. The circles outside of the polar plots represent the size of the error that was found for each specific subtask (see legend for scale). Only errors above the upper bound (as defined in Table 2) are shown

**Fig. 4:**
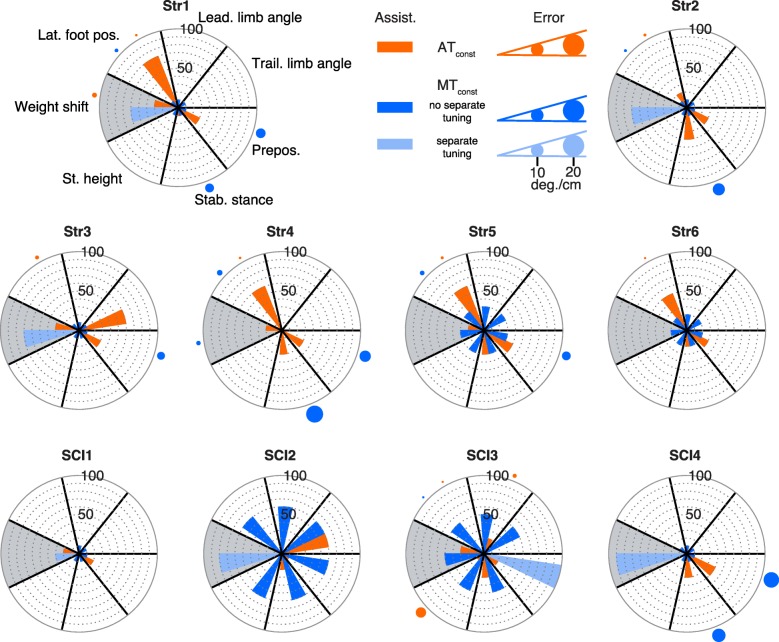
Assistance and errors for AT_const_ and MT_const_ for the less impaired leg (white background) and weight shift (grey background). Each polar plot shows the results for one participant. The distribution of the subtasks is the same for all polar plots (see Str1). The results for MT_const_ are split up into subtasks that were separately tuned by the therapist in a specific participant (light blue) and subtasks that were not separately tuned (dark blue). The bars represent the amount of assistance that the participants received for each specific subtask. The circles outside of the polar plots represent the size of the error that was found for each specific subtask (see legend for scale). Only errors above the upper bound (as defined in Table 2) are shown

Large differences in the assistance levels that were applied in AT_const_ and MT_const_ were found for both legs and the weight shift subtask (see Fig. 3 for the more impaired leg and Fig. 4 for the less impaired leg). The weight shift subtask is shown in both figures (Figs. 3 and 4, grey background), however, it is considered separately in the text below.

**Fig. 5:**
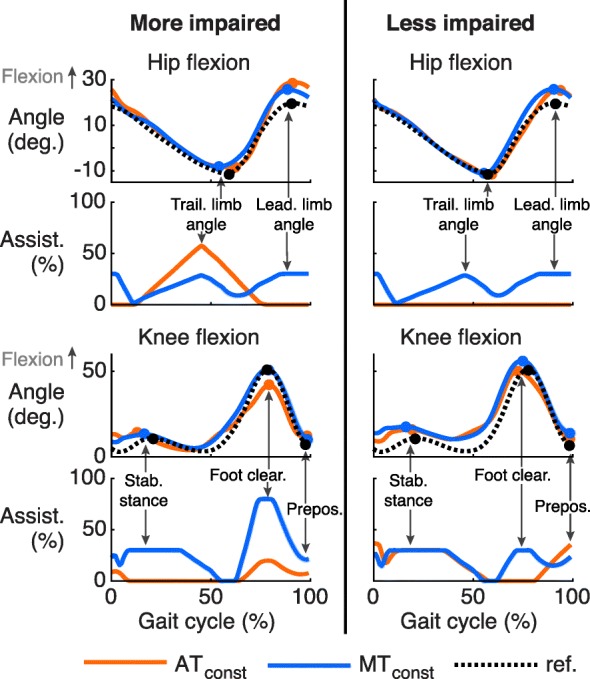
Average hip and knee flexion angles and assistance for Str5. Average angles and assistance across AT_const_ and MT_const_ are shown for Str5 for both legs as a function of gait cycle. The dots plotted on the trajectories indicate the evaluation points (see also Table 2) for the different subtasks

Figure 5 shows an example of the differences in assistance levels and the resulting joint trajectories for the knee and hip joints of Str5.

#### More impaired leg

For both approaches, AT and MT assistance, a higher assistance was applied for up to 4 specific subtasks of the more impaired leg in each participant, while less (MT) or no (AT) assistance was applied for other subtasks (see Fig. 3). Per participant, the therapist (MT approach) tuned 0 to 3 specific subtasks separately for the more impaired leg (see light blue bars in Fig. 3) while all other subtasks were (simultaneously) set to the same assistance level (dark blue bars). In 12 of the 60 cases (the term ’cases’ means subtasks for all participants (e.g. for the more impaired leg: 6 subtasks times 10 participants results in 60 cases)), the assistance for the more impaired leg was tuned separately by the therapist (see Table 4). In 11 of these 12 separately-tuned cases, the assistance was higher for MT_const_ compared to AT_const_ and for 1 of these 12 separately-tuned cases the same assistance was applied for both approaches. Also, for 33 of the 48 cases that were not tuned separately by the MT approach the assistance was higher in MT_const_ compared to AT_const_.

**Table 4:** Comparison of final assistance levels for MT and AT

	Separate tuning (MT)	No separate tuning (MT)
More impaired leg	12/60 cases	48/60 cases
MT >AT assist.	11/12	33/48
MT=AT assist.	1/12	1/48
MT <AT assist.	0/12	13/48
Less impaired leg	2/60 cases	58/60 cases
MT >AT assist.	1/ 2	34/58
MT=AT assist.	0/ 2	7/58
MT <AT assist.	1/ 2	17/58
Pelvis	6/10 cases	4/10 cases
MT >AT assist.	6/ 6	3/ 4
MT=AT assist.	0/ 6	0/ 4
MT <AT assist.	0/ 6	1/ 4

Cases means subtasks for all participants (e.g. for the more impaired leg: 6 subtasks times 10 participants results in 60 cases). The cases are split up into cases that were tuned separately by the therapist in MT_const_ and cases that were not separately tuned

**Fig. 6:**
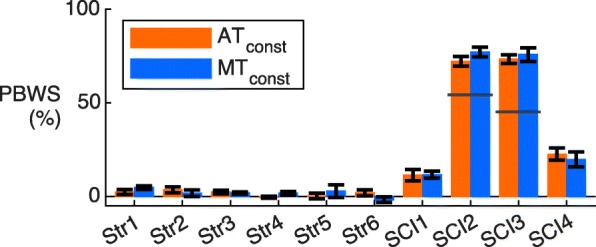
Partial body weight support. Average body weight support and standard deviation (between steps) for AT_const_ and MT_const_. The bars show the total PBWS (from the system and the use of the handrails). Only SCI2 and SCI3 received PBWS from the system (55% and 46%, respectively, indicated by the horizontal grey lines). All other PBWS is the result of using the handrails. Negative values can, for example, be explained by parts of LOPES II that might have slightly rested on the pelvis of the participant

Remarkably, for AT_const_, the most impaired participants (SCI2 and SCI3) did not receive much assistance (max. 40%) while these participants received at least 50% assistance for each subtask in MT_const_ (Fig. 3). These participants could probably walk with the low levels of assistance in AT_const_ due to the high levels of PBWS that were used (see Fig. 6). Although the same PBWS levels were applied for MT_const_ for SCI2 and SCI3 (and other participants, see Fig. 6), considerable differences were found for the assistance levels (Fig. 3). A possible reason for this is that the therapist was biased towards higher assistance levels due to the large impairments (i.e. low clinical scores) of SCI2 and SCI3 (Table 1). In addition, the therapist only knew the amount of PBWS provided by the system and he did not know the exact amount of PBWS as participants were using the hand rails for additional PBWS (see Fig. 6).

#### Less impaired leg

For the less impaired leg, deviations from the reference trajectories were such that AT_const_ resulted in assistance for up to 3 specific subtasks in each participant while the remaining subtasks did not receive any assistance (see Fig. 4). In contrast to this, with MT_const_ the assistance was not tuned separately in 58 of the 60 cases for the less impaired leg. For these 58 cases, the assistance applied by the MT approach was higher than the assistance applied by the AT approach in 34 cases (see Table 4).

#### Weight shift

The therapist (MT approach) separately changed the assistance for the weight shift in 6 of the 10 cases (see Figs. 3 and 4, grey background). In all of these separately-tuned cases, the weight shift assistance was higher for the MT approach compared to the AT approach (see Table 4). Also, for the other 4 cases (no separate tuning of weight shift by the therapist), the assistance was higher for the MT approach in 3 cases.

### Errors for final assistance levels

For both legs and the weight shift subtask, differences in the amount and magnitude of errors above the upper bound, which is the error at which assistance would be increased by the adaptive AT algorithm (see Table 2), were found.

#### More impaired leg

For the more impaired leg and MT_const_, the error was larger than the upper bound in 2 of the 12 cases that were tuned separately by the therapist (MT approach, see light blue dots in Fig. 3) and in 10 of the 48 cases that were not tuned separately (dark blue dots). For AT_const_, the error was larger than the upper bound in 10 of the 60 cases (orange dots). These errors for the AT algorithm were found because the algorithm did not adapt the assistance in AT_const_ and therefore, the assistance was not automatically increased when the error was larger than the upper bound.

Remarkably, although often less assistance was applied for AT_const_, the observed errors were much lower than for MT_const_ (always <10 deg.). The largest errors of up to 20 deg. of deviation from the reference trajectory were found for MT_const_, but only in subtasks that were not separately tuned by the therapist (e.g. stability during stance subtask (e.g. Str2, Str4) and prepositioning (SCI4), see Fig. 3).

#### Less impaired leg

For the less impaired leg only two subtasks were tuned separately by the therapist and for these subtasks the error was lower than the upper bound. For 24% of the 58 subtasks that were not tuned separately, the error was larger than the upper bound (see dark blue dots in Fig. 4). For AT_const_, the error was larger than the upper bound in only 9 of the 60 cases (orange dots).

The largest errors (up to 18 deg. of deviation from the reference trajectory) were found for MT_const_ for the stability during stance and prepositioning subtasks (see Fig. 4). In most cases, the errors for AT_const_ were much lower. Only for one of the participants (SCI3) an error of 10 deg. was found for the foot clearance subtask in AT_const_, while all other errors were smaller than 10 deg.

#### Weight shift

Resulting errors for the weight shift subtask were generally small. Separate tuning of the weight shift subtask in MT_const_ always resulted in errors lower than the upper bound (see Figs. 3 and 4). Only in one case the error was higher than the upper bound in MT_const_ when the assistance was not selectively tuned. The AT algorithm also resulted in errors lower than the upper bound in all except for one participants. The error was less than 5 cm in both cases (MT_const_ and AT_const_).

### Questionnaires

#### Participants’ preferences

**Fig. 7:**
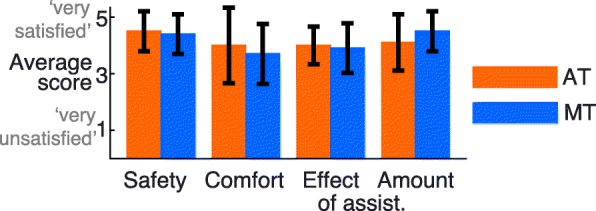
Average evaluation of the AT and MT algorithms by all participants. The aspects safety, comfort, effect of assistance and amount of assistance were evaluated on a scale from 1 (very unsatisfied) to 5 (very satisfied). The average for all participants and standard deviation between participants is shown

Participants evaluated safety, comfort and effect and amount of assistance on a scale with a maximum of 5. On average, participants gave similar (high) scores for the safety (AT: 4.5, MT: 4.4) and the effect of assistance (AT: 4.0, MT: 3.9) (see Fig. 7). The comfort was evaluated slightly better for the AT algorithm (4.0) compared to MT assistance (3.7). In contrast to this, participants were slightly more satisfied with the amount of assistance given by the MT algorithm (4.5) compared to the AT algorithm (4.1). The scores per participant were also checked to see whether there were clear differences between the two approaches in specific participants, however, the difference between AT and MT assistance was never larger than 1 for any of the questions.

#### Therapist

The therapist answered in eight of the ten participants that he/she adjusted the assistance for specific subtasks separately. The therapist decreased the assistance for all other subtasks to assist the most impaired subtasks, but let the participants do as much as possible by themselves. For four of the ten participants the therapist was satisfied with the result. For the other participants he/she was not satisfied with the exact effect of the assistance. Besides, the therapist claimed that it was often difficult to see what exactly changed (e.g. when decreasing the assistance), and that he/she sometimes had to rely on feedback from the participants.

## Discussion

The goal of this study was to compare subtask-based MT and AT robotic assistance during gait in people with neurological disorders. We determined differences while tuning the assistance, final assistance levels, errors compared to reference trajectories and preferences of the participants. For all of these aspects, large differences were found between the AT and MT approach, except for the preferences of the participants, which were similar for both approaches.

### Possible reasons for differences between the AT and MT approach

There might be several reasons for the large differences in final assistance levels (and deviations from the reference trajectories) between the two approaches that can only be speculated on. The AT algorithm assured a good performance for all subtasks by tuning the assistance for each subtask separately. In contrast to this, the therapist (MT approach) tuned a small number of subtasks separately (the most affected ones) and aimed for a good performance (low errors) for these subtasks. For the subtasks that were not tuned separately, the largest errors were found, which means that the therapist accepted larger deviations for these subtasks. Although the therapist could have used the GUI to see the deviations from reference trajectories for all subtasks, he/she was mainly relying on visual assessment of the gait pattern and feedback from the participants when tuning the assistance. This could be an indication that the therapist did not attempt to decrease the deviations from the reference trajectories for all subtasks, but rather tried to reach an acceptable walking pattern. In addition, the therapist might have accepted larger deviations from reference trajectories to allow for compensation strategies.

Another possible reason for the differences between the AT and MT approach is that tuning all subtasks separately could be too complicated and time-consuming for clinical practice. The subtasks were related to common problems after neurological disorders [36–40]. They were chosen based on input from physical therapists and rehabilitation physicians who indicated that they would like to have more possibilities to tune the assistance than in other (commercially available) robotic gait trainers, which often only allow to change the general assistance for the whole gait cycle and multiple joints simultaneously [4, 32]. The number of subtasks in the current study is relatively low (6 for each leg, and weight shift). Still, in this study, the therapist focused only on a low number of subtasks (up to 4 per participant) and tuned these subtasks separately.

A last possible reason for the difference between the AT and MT approach is that the therapist might also have acted on the safe side, by trying to prevent possible problems occurring with (too) low assistance levels (e.g. stumbling, exhaustion) and therefore more often higher assistance levels were found for the MT approach. An indication for this could be that for the most impaired participants the MT assistance was much higher than needed, even for most subtasks that were not tuned separately.

### Advantages of the AT approach compared to the MT approach

A large advantage of the AT approach is that it is not influenced by subjective decisions of the therapist. However, there are various other factors that can be used to determine whether the AT or MT approach is better. In this study, we focused on the time to tune the assistance, the amount of assistance and deviations from reference trajectories.

The time to tune the assistance is an important factor that needs to be considered for clinical application. If the tuning takes too long, patients might not be able to exercise at their desired assistance levels as they might be too fatigued or the training session might end before the desired assistance levels are reached. In our study, the AT algorithm reached a constant assistance level more quickly than the MT algorithm. Two studies with other AT algorithms also have shown that stable assistance levels can be reached within a similar time as in our current study with an automatic algorithm [13, 21].

Another factor that we considered was the amount of assistance. From literature, it is known that active participation is an important factor in rehabilitation after neurological disorders and applying too much assistance might hinder recovery [4, 41–43]. There is accumulating evidence that focusing on algorithms that tailor therapy to the patient’s needs by only applying as much assistance as needed, can increase training intensity and improve outcomes of RAGT. For example, Srivastava et al. [11] and Krishnan et al. [33] have shown that AT algorithms for RAGT can lead to improvements in clinical scales, however, no control groups were included in these studies to compare the AT algorithms to other approaches. Park et al. [44] found that progressively reducing the amount of assistance from 100% to 60% can lead to larger improvements in FAC score and Berg balance scale in people with subacute stroke compared to applying 100% assistance during a training program of four weeks. Though the evidence is still preliminary, these studies indicate that personalized and reduced robotic assistance leads to larger improvements. In this regard, better results were obtained for the AT algorithm in our current study: every subtask was tuned separately and the assistance was more often lower for the AT approach than for the MT approach.

Even though less assistance was often applied by the AT algorithm, the largest deviations from the reference trajectories were found for the MT approach. It is debatable how closely measured trajectories need to match reference trajectories (i.e. physiological trajectories) in RAGT as allowing compensatory mechanisms might also be beneficial [21, 42]. In the current study, the AT approach resulted in walking patterns close to the reference trajectories and assistance might have been increased to prevent compensatory strategies. In contrast to this, the therapist could have allowed compensatory strategies by decreasing assistance. In the future, the MT approach might be more suitable when compensatory strategies should be allowed, while the AT approach leads to smaller errors in the evaluation points (i.e. more physiological gait pattern).

Next to the factors that were analyzed in this study (time to tune the assistance, amount of assistance and deviations from reference trajectories), there are more factors that could influence the therapeutic effect of RAGT. For example, it is not known yet if assisting a specific subtask might lead to better clinical outcomes than assisting another specific subtask. In addition to this, applying less assistance might be more exhausting and result in shorter training duration (although fatigue might be partly compensated for by automatically increasing assistance with the AT algorithm). It is not known yet how shorter (but more intensive) robotic gait training sessions would affect therapy outcomes compared to longer (less intensive) training sessions [2].

To sum up, regarding the time to tune the assistance, the amount of assistance and deviations from reference trajectories, the AT algorithm has more advantages than the MT approach. However, we cannot draw any decisive conclusions about possible clinical outcomes yet as there are too many factors that might affect clinical outcomes.

### Study limitations

Deriving reference trajectories for robot-assisted gait training is crucial but difficult. We used reference trajectories that depended on walking speed and body length [34]. However, these trajectories were collected during treadmill walking and did not take into account that the dynamics of the robot or PBWS could influence the gait pattern [15]. It is still debated whether reference trajectories should be adjusted based on robot dynamics, PBWS or other therapeutic goals. In our current study, when using trajectories based on treadmill walking that were not adjusted to the specific gait trainer, maximal hip flexion was larger than the reference trajectory for nearly all participants (for the AT and MT approach). Therefore, the assistance that was applied for the leading limb angle subtask (mainly for the MT approach) might have impeded motion and decreased maximal hip flexion. Having the option to automatically (e.g. based on less impaired leg) or manually [10] change the reference trajectories might be useful for future training protocols.

Another limitation is that only one experienced therapist tuned the assistance in this study. For example, there might be differences in the settings that are applied by an experienced therapist compared to an inexperienced therapist (or compared to another experienced therapist). To our knowledge, there are no studies that compare the assistance that is applied by an experienced and inexperienced therapist for RAGT, especially not for LOPES II. Still, other studies analyzed differences between therapists for physical assistance that was applied during training. In [45], seven therapists applied similar forces to correct balance in stroke survivors during overground training. However, Galvez et al. [46] showed that the physical assistance applied to the legs of SCI patients during body weight supported treadmill training was different between experienced and inexperienced therapists. It is not clear yet what the exact reasons for the differences were and if they would also appear for tuning of RAGT. However, as various settings can be changed in LOPES II (six subtasks per leg, and weight shift) and the therapist in the current study was mainly relying on (subjective) visual assessment of the gait pattern, we would expect differences between therapists, especially between novice users and experienced therapists. Therefore, in future studies, experiments should be performed with multiple therapists (experienced and inexperienced) and/or therapists should be taught to rely more on the objective and quantitative feedback that is provided by the GUI as it is expected that this will lead to lower variability between therapists.

### Future directions

Instead of choosing for either AT or MT assistance, in the future, a combined AT and MT approach might be used to take advantage of both approaches. Some possibilities that could be investigated in future studies are:

(1) The AT algorithm could be used to give recommendations on the amount of assistance to apply while the therapist still has to take the final decision about which assistance levels are applied. The advantage of this is that the therapist’s knowledge is taken into account, he/she has control over the training, he/she can take into account feedback from the patient and the AT algorithm might show that the user needs more assistance on certain subtasks that the therapist might not have taken into account otherwise. A disadvantage is that tuning of the assistance might be slower than with an AT algorithm alone.

(2) The assistance for all subtasks is AT, however, the therapist could choose to tune some specific subtasks manually if he/she does not agree with the effect of the AT algorithm or wants to reduce specific errors even more. This would still give the therapist some control, the therapist could take into account feedback from the patient, but it would also make the whole process quicker as the therapist would not have to tune the exact assistance levels for each subtask anymore. Besides, compared to MT assistance alone which could be focused on a low number of subtasks, all subtasks would be tuned to the specific needs of the patient.

(3) Another possibility would be that the therapist chooses more discrete levels (e.g. low, medium, high) which are each associated with a specific range of assistance levels (e.g. low from 0-30%). Within these discrete levels an AT algorithm could choose the exact amount of assistance. In this case, the therapist would still be able to choose a broad assistance level based on his/her experience and feedback from the patient, and he/she is assisted by the AT algorithm in quickly choosing the exact level of assistance.

Although it is not known which combination would work best, we believe that a combination of AT and MT subtask-based assistance could be beneficial for future RAGT as it would take into account therapist’s knowledge and experience, it allows the patient to give feedback, but it also simplifies tuning of the parameters compared to MT assistance alone.

In addition, it should be investigated whether the AT algorithm itself can be further improved. To promote active participation of the patient, our AT algorithm decreases the assistance when errors are small, however, it is not known yet whether adding a forgetting factor [14, 16] leads to even more active participation of the patient. It might also be beneficial to automatically tune other parameters (e.g. PBWS, walking speed) as these can also affect the gait pattern and amount of assistance that is applied by an AT algorithm [15].

## Conclusions

We have found large differences in the assistance applied by an automatically-tuned and manually-tuned algorithm. Advantages of the AT approach compared to the MT approach were that the assistance was tuned quicker, lower assistance levels were used (enhancing active participation of the user), each subtask was tuned separately and a good performance was assured for all subtasks. In contrast to this, the MT approach focused on a limited number of subtasks (two to four) that were tuned separately. Future clinical trials need to show whether these apparent advantages of the AT approach result in better clinical outcomes. To exploit the advantages of the AT approach (e.g. quick tuning of all subtasks) and take into account the experience of therapists and feedback from patients during the training, a combined approach of manual and automatic tuning should be considered in the future.

The results from this study can be used to develop more extended (clinical) studies that are needed to get insight into the long-term effect of AT and MT subtask-based training protocols on walking function after neurological disorders.

## Data Availability

The datasets generated and/or analyzed during the current study are available from the corresponding author on reasonable request.

## Abbreviations

10MWT; 10 meter walking test; AFO: Ankle foot orthosis
AT: Automatically-tuned
DOF: Degree of freedom
FAC: Functional ambulation scale
FMA: Fugl-Meyer assessment
GUI: Graphical user interface
LOPES: Lower extremity power exoskeleton
MI: Motricity index
MT: Manually-tuned
PBWS: Partial body weight support RAGT: Robot-assisted gait therapy
SCI: Spinal cord injury
